# Solubility of dicarbohydrazide bis[3-(5-nitroimino-1,2,4-triazole)] in common pure solvents and binary solvents at different temperatures

**DOI:** 10.1098/rsos.190728

**Published:** 2019-11-13

**Authors:** Jianrong Ren, Dong Chen, Yanwu Yu, Hongzhen Li

**Affiliations:** 1 Institute of Chemical Materials, China Academy of Engineering Physics, Mianyang, Sichuan 621900, People's Republic of China; 2 College of Environment and Safety Engineering, North University of China, Taiyuan 030051, People's Republic of China

**Keywords:** thermodynamics, DCBNT, energetic ionic salt, solubility

## Abstract

The solubility of dicarbohydrazide bis[3-(5-nitroimino-1,2,4-triazole)] (DCBNT) was first measured under the different pure solvents and binary solvents by the dynamic method over the temperature range of 290–360 K at atmospheric pressure. Results in all the solvents were positively correlated with temperature, namely increased with increasing temperature. The experiment data were correlated by the Apelblat equation, the Yaws equation and the polynomial equation. The conclusion showed that these three models all agreed well with the experimental data. Simultaneously, the dissolution enthalpy, dissolution entropy and Gibbs free energy of DCBNT in different solvents were calculated from the solubility data by using the Apelblat model. The results indicate that the dissolution process of DCBNT in these solvents is driven by entropy, which provides theoretical guidance for further research on the crystallization of DCBNT.

## 1. Introduction

Solubility evaluation plays a significant role in the purification and separation process in the industry of chemical production. It is well known that the density, energy, safety and compatibility with other chemicals of explosives are closely related to their crystal purity, particle size and morphology. In particular, the particle morphology of explosives was found to have important impact on its safety and energy performance [1]. Therefore, in order to obtain high-quality and high-performance crystals, it is very important to design a reliable crystallization process and optimize the crystallization conditions in solvents to control the crystallization quality. The solubility data of compounds are important to control and optimize the crystallization process, since it will determine the selection of the crystallization method and the crystallization solvents [2–6]. On the other hand, thermodynamic parameters (dissolution enthalpy and entropy) can provide considerable information about the dissolving process of compounds in solvents, such as the endothermic or exothermic, entropy-driven and enthalpy-driven processes [7].

**Figure 1.**
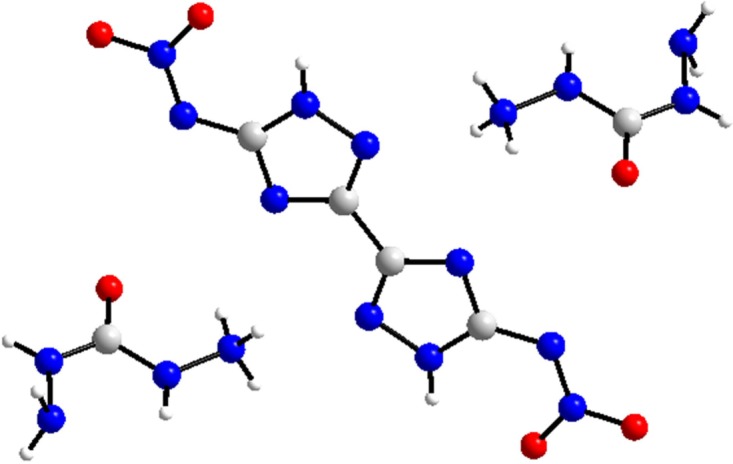
Molecular structure of dicarbohydrazide bis[3-(5-nitroimino-1,2,4-triazole)].

Nowadays, as alternatives to high-performance energetic materials, energetic ionic salts (EISs) have attracted increasing attention [8], especially for their lower vapour pressures, higher positive heats of formation, better thermal stability and higher densities than the atomically similar non-ionic compounds [8–10]. Dicarbohydrazide bis[3-(5-nitroimino-1,2,4-triazole)] (DCBNT) [11] (figure 1) is a novel EIS, with a moderate density of 1.780 g cm^−3^, a high detonation velocity of 9234.87 m s^−1^ and a detonation pressure of 31.73 GPa, which is calculated by EXPLO5 v. 6.02. Besides, DCBNT exhibits good thermal stability, as the decomposition peak temperature is over 230°C. Its impact sensitivity is greater than 40 J, and the friction sensitivity is 216 N. The high thermal stability, low sensitivity towards impact and friction as well as the good detonation properties make DCBNT a potential kind of low-sensitive and high-energetic explosive [12].

In this study, we tested the solubility of DCBNT in 12 commonly used solvents: water (H_2_O), dimethyl sulfoxide (DMSO), *N*,*N*-diethylformamide (DEF), *N*,*N*-dimethylformamide (DMF), 1,4-butyrolactone (BL), methanol, ethanol, acetone, trichloromethane, dioxane, acetonitrile and ethyl acetate, and five binary solvents (volume ratio = 1 : 1), at atmospheric pressure using a polythermal method [13,14] with the CrystalSCAN system. The experimental solubility data were correlated by the modified Apelblat model, the Yaws model and the polynomial model. The thermodynamic magnitudes, such as the dissolution enthalpy, dissolution entropy and molar Gibbs free energy, were then obtained from the solubility data. The driving force of the process was determined by enthalpy–entropy compensation analysis [15].

## 2. Experimental

### 2.1. Materials

**Figure 2.**
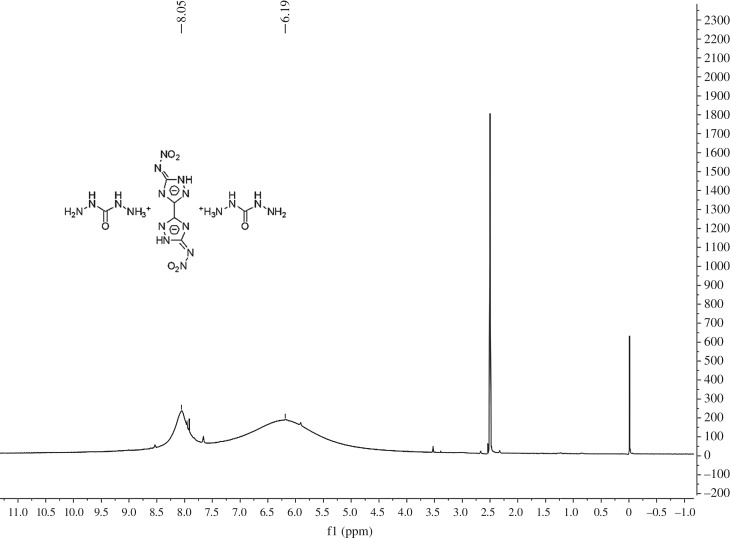
^1^H NMR spectrum of DCBNT.

**Figure 3.**
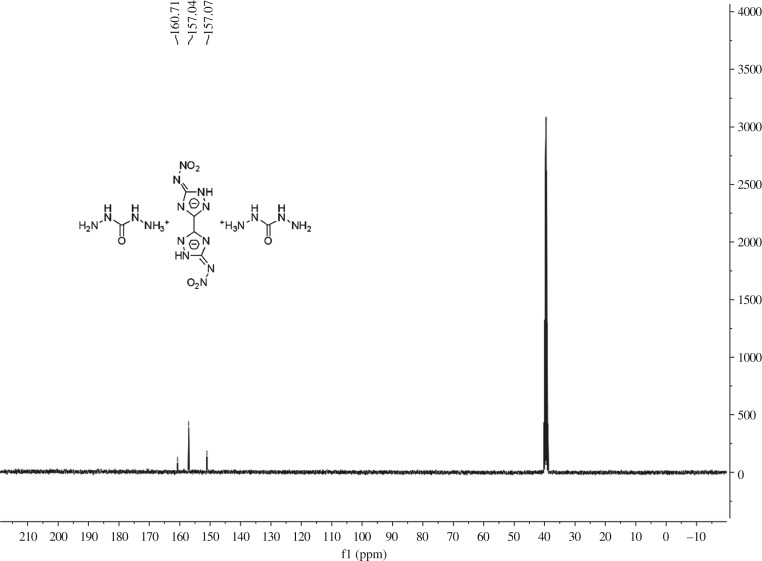
^13^C NMR spectrum of DCBNT.

DCBNT [11] was synthesized by our research group according to Shreeve and co-workers [16]. The purity of DCBNT, 99.27%, was determined by high-performance liquid chromatography [17]. Distilled water was prepared in our laboratory and used throughout. All reagents were purchased commercially and used without further purification. The ^1^H NMR and ^13^C NMR are shown in figures 2 and 3, respectively.

### 2.2. Apparatus

**Figure 4.**
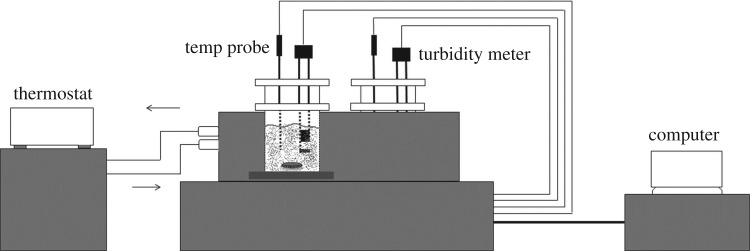
Schematic of the HEL CrystalSCAN system for solubility measurement.

The solubility data of DCBNT were measured by the dynamic method and collected by the CrystalSCAN system (E1320, HEL Ltd, UK; figure 4). The mass of DCBNT was weighed using an analytical balance (CP225D, Sartorius, Germany) with an accuracy of 10^−4^ g. Circulating oil solution from a thermostat (Huber CC1-505wl vpc55, Germany) used with an uncertainty of *u*(*T*) = 0.01 K controlled the temperature of the mixture. ^1^H and ^13^C spectra were recorded on a 400 MHz (Bruker AVANCE 400) or 600 MHz (Bruker AVANCE 600) nuclear magnetic resonance spectrometer.

### 2.3. Solubility determination

The solubility of DCBNT in all the solvents was tested by the dynamic method with a turbidity explorer. A known amount of DCBNT was added to an appropriate glass vial with 60 ml of solvent, the solution was then slowly heated at a specific speed and kept stirred, and the dissolved ability was judged by the turbidity curve. The heating rate was 0.2 K min^−1^ and the stirring rate was 500 r.p.m. With temperature increasing, the turbidity changed gradually. When turbidity reached its minimum and remained unchanged for a long time, representing a full dissolution, this dissolution temperature was recorded as *T*. In order to reduce the deviation, each experiment was performed three times, and the average of three measurements was determined as the final value. The estimated relative standard uncertainty of the temperature was less than 0.003. The mole fraction solubility (*x*) of DCBNT in different pure solvents can be calculated by the following equation [18]:

2.1 $$x=\frac{m_{1}/M_{1}}{m_{1}/M_{1}+m_{2}/M_{2}},$$

where *M*_1_ and *M*_2_ are the molecular masses of DCBNT and solvent, respectively; *m*_1_ and *m*_2_ represent the corresponding mass of DCBNT and solvent, respectively.

The calculation method for the mole fraction solubility (*x*) of DCBNT in binary solvents is the same as that of DBNT in pure solvent [19].

2.2 $$x=\frac{m_{1}/M_{1}}{m_{1}/M_{1}+m_{3}/M_{3}+m_{4}/M_{4}},$$

where *M*_1_, *M*_3_ and *M*_4_, and *m*_1_, *m*_3_ and *m*_4_ present the molecular masses and the masses of DCBNT, organic solvent and water, respectively.

## 3. Solubility models

All the solubility data obtained from pure solvents and binary solvents at different temperatures were correlated by three models: modified Apelblat model [20], Yaws model [21,22] and polynomial model, which were widely used.

### 3.1 Modified Apelblat model

The relationship between mole fraction solubility and temperature can be described by the Apelblat model. The expression is shown in the following equation:

3.1 $$\text{ln}x=A_{1}+\frac{B_{1}}{T}+C_{1}\ln T,$$

where *x* is the mole fraction solubility of DCBNT and *T* is the absolute temperature (K). *A*_1_, *B*_1_ and *C*_1_ are the empirical model parameters. They can be obtained to fit the experimental data by a nonlinear least-squares method [23].

### 3.2 Yaws model

For the Yaws model, the relationship between mole fraction solubility and temperature can be described as follows:

3.2 $$\text{ln}x=A_{2}+\frac{B_{2}}{T}+\frac{C_{2}}{{\left(T\right)}^{2}},$$

where *x* is the mole fraction solubility of DCBNT; *T* is the absolute temperature (K) and *A*_2_, *B*_2_ and *C*_2_ are the empirical parameters of the model. They can be obtained to fit the experimental data by the nonlinear least-squares method.

### 3.3. Polynomial model

The relationship between mole fraction solubility of DCBNT and temperature was also correlated with the polynomial model. The specific expressions are as follows:

3.3 $$x=A_{3}+B_{3}T+C_{3}T^{2},$$

where *x* is the mole fraction solubility of DCBNT; *T* is the absolute temperature (K) and *A*_3_, *B*_3_ and *C*_3_ are the empirical parameters of the model.

## 4. Results and discussion

### 4.1. Solubility data

**Figure 5.**
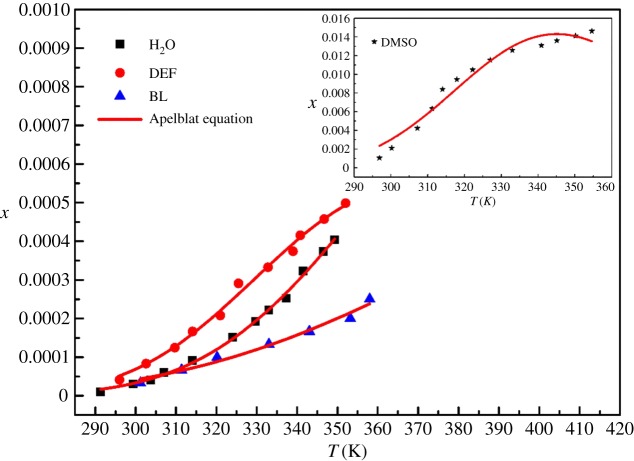
Mole fraction solubility *x* of DCBNT in different solvents: (⋆) DMSO; (▪) H_2_O; (●) DEF; (▴) BL. The line is the best fit of the experimental data calculated with the Apelblat equation.

**Figure 6.**
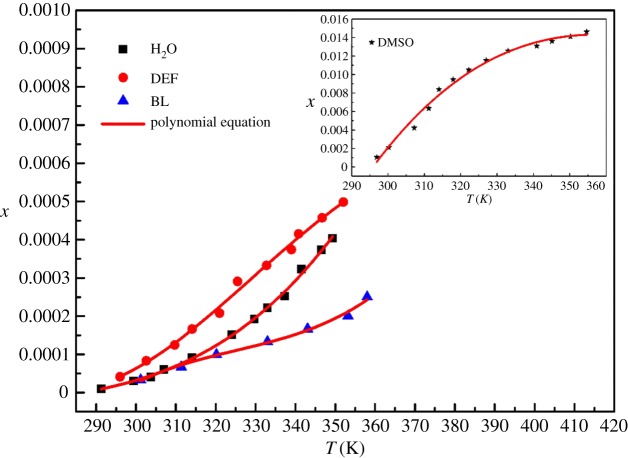
Mole fraction solubility *x* of DCBNT in different solvents: (⋆) DMSO; (▪) H_2_O; (●) DEF; (▴) BL. The line is the best fit of the experimental data calculated with the polynomial equation.

**Figure 7.**
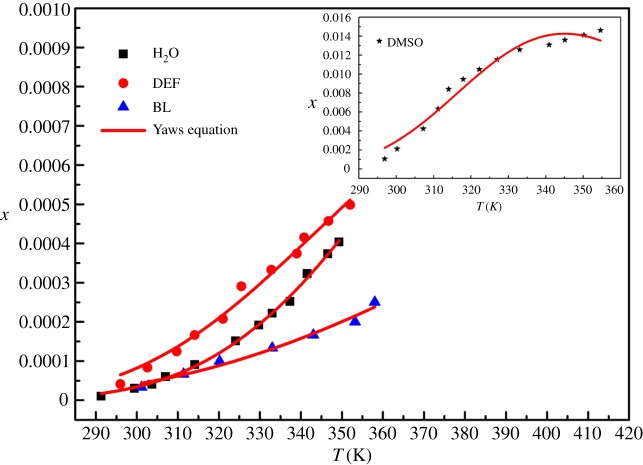
Mole fraction solubility *x* of DCBNT in different solvents: (⋆) DMSO; (▪) H_2_O; (●) DEF; (▴) BL. The line is the best fit of the experimental data calculated with the Yaws equation.

It was found through experiments that the DCBNT is almost insoluble in most solvents, including DMF, methanol, ethanol, acetone, chloroform, dioxane, acetonitrile and ethyl acetate. On the other hand, DCBNT has better solubility in DMSO, H_2_O, DEF and BL at temperatures from 290 to 360 K, and they are listed in table 1 and shown in figures 5–7. It can be found that the solubility of DCBNT in these selected pure solvents increased with increasing temperature. The solubility of DCBNT in DMSO is much higher than that in the other three solvents. Moreover, the order of DCBNT solubility in different solvents is: DMSO > DEF > H_2_O > BL, by further comparing the four sets of data, and it can also be seen that the mole fraction solubility of DCBNT in DMSO is nearly 100 times higher than that in H_2_O. According to the principle of ‘like dissolves like’ [24,25], the solubility of DCBNT in H_2_O should be better than that in DMSO, so the solubility of DCBNT may not only depend upon the solvent polarity but also upon other factors. Although the solubility of DCBNT in H_2_O, DEF and BL is not so good as in DMSO, the solubility curve changes obviously with temperature, so it can also be used as an alternative solvent for cooling crystallization of DCBNT.

**Table 1.** Mole fraction solubility *x* of DCBNT in pure solvents at different temperatures under 101 kPa^a^.

		Apelblat model		polynomial model		Yaws model	
*T* (K)^b^	1000*x*^c^	1000*x*^cal^	RD	1000*x*^cal^	RD	1000*x*^cal^	RD
DMSO							
296.9	1.06	1.95	−0.840	0.88	0.170	1.89	−0.783
301.2	2.52	2.75	−0.091	2.45	0.028	2.71	−0.075
307.2	4.22	4.16	0.014	4.58	−0.085	4.15	0.017
312.2	6.02	5.56	0.076	6.19	−0.028	5.59	0.071
317.9	7.85	7.35	0.064	7.89	−0.005	7.40	0.057
325.2	9.61	9.71	−0.104	9.81	−0.021	9.75	−0.015
333	11.82	11.98	−0.014	11.56	0.022	11.98	−0.014
340.9	13.38	12.43	0.071	12.98	0.030	13.54	−0.012
348.1	13.99	14.23	−0.017	14.00	−0.001	14.2	−0.015
354.6	14.42	14.09	0.023	14.68	−0.018	14.14	0.019
H_2_O							
291.3	0.010	0.017	−0.700	0.015	−0.500	0.017	−0.700
299.4	0.030	0.032	−0.067	0.029	0.033	0.032	−0.067
303.7	0.040	0.044	−0.100	0.041	−0.025	0.044	−0.100
307	0.060	0.055	0.083	0.053	0.117	0.055	0.083
314.1	0.091	0.086	0.055	0.087	0.044	0.086	0.055
324.1	0.151	0.148	0.020	0.152	−0.001	0.148	0.020
329.7	0.192	0.193	−0.005	0.196	−0.021	0.193	−0.005
333	0.222	0.224	−0.010	0.226	−0.018	0.224	−0.009
337.3	0.252	0.267	−0.060	0.268	−0.063	0.266	−0.056
341.5	0.323	0.312	0.034	0.312	0.034	0.312	0.034
346.5	0.374	0.372	0.005	0.370	0.011	0.371	0.008
349.3	0.404	0.406	−0.005	0.404	0	0.406	−0.005
DEF							
296	0.042	0.052	−0.238	0.034	0.190	0.050	−0.190
302.6	0.083	0.083	0	0.082	0.012	0.082	0.012
309.7	0.125	0.127	−0.016	0.137	−0.096	0.127	−0.016
314.1	0.166	0.161	0.030	0.171	−0.030	0.161	0.030
321	0.208	0.221	−0.063	0.227	−0.091	0.222	−0.067
325.5	0.291	0.264	0.093	0.266	0.086	0.265	0.089
332.8	0.332	0.335	−0.009	0.329	0.009	0.336	−0.012
339	0.374	0.394	−0.053	0.385	−0.029	0.394	−0.053
340.8	0.416	0.411	0.012	0.401	0.036	0.410	0.014
346.7	0.457	0.458	−0.02	0.456	0.002	0.458	−0.002
352	0.499	0.493	0.012	0.507	−0.016	0.494	0.010
BL							
301.2	0.033	0.044	−0.333	0.038	−0.152	0.044	−0.333
311.4	0.067	0.066	0.015	0.065	0.030	0.065	0.030
320.2	0.100	0.089	0.110	0.091	0.09	0.088	0.120
333	0.133	0.131	0.015	0.134	−0.008	0.131	0.015
343	0.167	0.170	−0.018	0.172	−0.030	0.171	−0.023
353.2	0.200	0.215	−0.075	0.215	−0.075	0.215	−0.075
358	0.250	0.238	0.048	0.236	0.056	0.238	0.048

^a^Standard uncertainties *u* are *u*(*T*) = 0.01 K, *u*(*P*) = 3 kPa.

^b^The estimated relative standard uncertainty of the temperature is *ur*(*T*) = 0.003.

^c^*x* is the experimental solubility data of DCBNT.

The comparison between the calculated and experimental values is shown in table 1. The relative deviation (RD) is given in table 1. The regression parameters of each model are given in table 2. In addition, we calculated the relative average deviation (RAD) and root-mean-square deviation (RMSD), which are important for evaluating the applicability and accuracy of the models used in this study. RD is shown in the following equation:

4.1 $$\text{RD}=\frac{x_{i}^{\exp}-x_{i}^{\mathrm{cal}}}{x_{i}^{\exp}}.$$

**Table 2.** Regression parameters, RAD and RMSD for the solubility of DCBNT in pure solvents with different models at pressure *p* = 101 kPa^a^.

equation	solvent	A^b^	B^b^	C^b^	*R*^2^^c^	RAD^d^	RMSD^e^
Apelblat model	DMSO	947.879	−48 593.720	−138.836	0.9907	0.131	4.9 × 10^−4^
	H_2_O	394.398	−23 740.123	−57.077	0.99733	0.095	6.37 × 10^−6^
	DEF	632.005	−34 044.067	−92.588	0.99225	0.050	1.17 × 10^−5^
	BL	134.809	−9677.480	−19.746	0.97239	0.088	9.45 × 10^−6^
polynomial model	DMSO	−0.349	0.002	−2.642 × 10^−6^	0.9967	0.041	2.31 × 10^−4^
	H_2_O	0.008	−5.837 × 10^−5^	1.016 × 10^−7^	0.9970	0.072	6.57 × 10^−6^
	DEF	−1.025 × 10^−4^	−6.254 × 10^−6^	2.268 × 10^−8^	0.9915	0.010	1.22 × 10^−5^
	BL	9.947 × 10^−4^	−8.780 × 10^−6^	1.861 × 10^−8^	0.9763	0.063	8.92 × 10^−6^
Yaws model	DMSO	−65.849	43 203.612	−7.576	0.9922	0.010	3.62 × 10^−4^
	H_2_O	−22.062	13 804.300	−3.083	0.9974	0.095	6.21 × 10^−6^
	DEF	−43.986	27 144.441	−5.048	0.9926	0.004	1.15 × 10^−5^
	BL	−10.326	4106.828	−1.84	0.9729	0.092	9.70 × 10^−6^

^a^The standard uncertainty is *u*(*P*) = 3 kPa.

^b^A, B and C are parameters of different models.

^c^*R*^2^ stands for correlation coefficient.

^d^RAD is the average relative deviation.

^e^RMSD represents the root-mean-square deviation.

The RAD is described as follows:

4.2 $$\text{RAD}=\frac{1}{N}\overset{N}{\underset{i=1}{\sum }}\left|\frac{x_{i}^{\exp}-x_{i}^{\mathrm{cal}}}{x_{i}^{\exp}}\right|.$$

The RMSD is defined as follows:

4.3 $$\text{RMSD}=\sqrt{\frac{\sum_{i=1}^{N}{\left(x_{i}^{\exp}-x_{i}^{\mathrm{cal}}\right)}^{2}}{N}}.$$

In equations (4.1)–(4.3), $x_{i}^{\exp}$ and $x_{i}^{\mathrm{cal}}$ represent the experimental and computational values of molar fractional solubility of DCBNT, respectively. *N* represents the number of points measured in the experiment.

As can be seen from figures 5–7, the experimental data are basically consistent with the empirical equation data, and the experimental data are evenly distributed near the fitting line. The closer the *R*^2^ value is to 1, the higher the reference value of the empirical equation. From tables 1 and 2, we can find that the values of correlation coefficient (*R*^2^) are all close to 1, which indicates that the values obtained by the three models are in good agreement with the experimental values, especially in DMSO, DEF and H_2_O. In addition, we also find that the Apelblat model is better than the polynomial model and the Yaws model in correlating solubility data in DMSO and BL. For DEF and H_2_O, the Yaws model is better than the Apelblat equation and the polynomial model. Moreover, the RADs and RMSDs obtained by fitting the solubility data of DCBNT in four pure solvents by the three models are not very different. In terms of RMSD, it will be found that the values of DMSO, H_2_O, DEF and BL (2.31 × 10^−4^, 6.57 × 10^−6^, 1.22 × 10^−5^ and 8.92 × 10^−6^) correlated with the polynomial model are slightly better than those fitted by the Apelblat model (4.9 × 10^−4^, 6.37 × 10^−6^, 1.17 × 10^−5^ and 9.45 × 10^−6^) and the Yaws model (3.62 × 10^−4^, 6.21 × 10^−6^, 1.15 × 10^−5^ and 9.70 × 10^−6^), which shows that the calculated values obtained by the polynomial method are less deviated from the experimental values. In sum, all three models are suitable for describing the solubility of DCBNT in the selected pure solvents.

**Figure 8.**
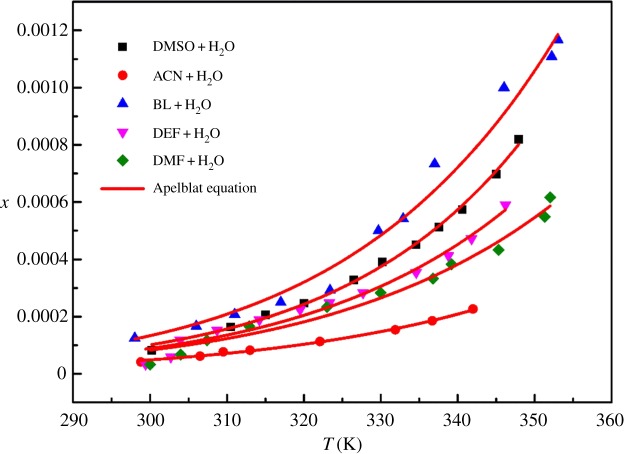
Mole fraction solubility *x* of DCBNT in binary solvents. The line is the best fit of the experimental data calculated with the Apelblat equation.

**Figure 9.**
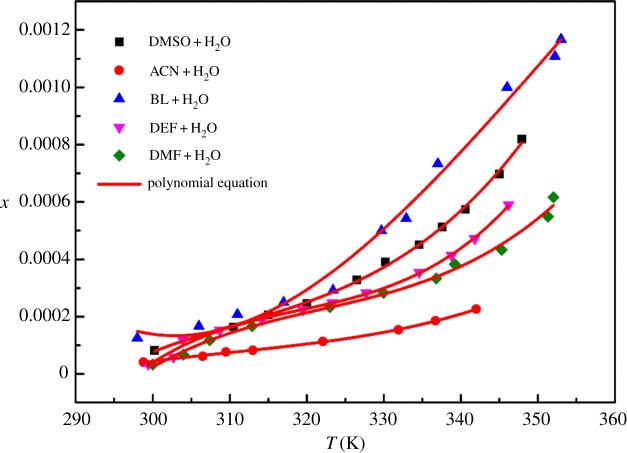
Mole fraction solubility *x* of DCBNT in binary solvents. The line is the best fit of the experimental data calculated with the polynomial equation.

**Figure 10.**
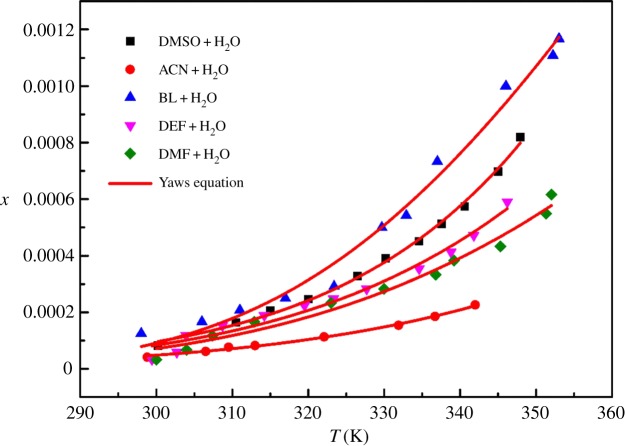
Mole fraction solubility *x* of DCBNT in different binary solvents. The line is the best fit of the experimental data calculated with the Yaws equation.

In the crystallization process, when the solubility of compounds in pure solvents is low, recrystallization with mixed solvents is a common method. The solubility of DCBNT in different binary solvents was also tested in the range of 290–360 K. The results made clear that the solubility of DCBNT in acetone + H_2_O is abnormal (the experimental data fluctuate greatly, it is probably because acetone evaporates too quickly) and almost insoluble in methanol + H_2_O and ethanol + H_2_O. Therefore, the available mixed solvents include BL + H_2_O, DMSO + H_2_O, DEF + H_2_O, DMF + H_2_O and ACN + H_2_O. The results are shown in table 3. The solubility data in these five binary solvents (volume ratio = 1 : 1) are also correlated by the Apelblat model equation, the Yaws model and the polynomial model equation (equations (3.1)–(3.3)), and the values of the parameters are listed in table 4. The RADs and RMSDs are also given in table 4. Figures 8–10 are curves of the mole fraction solubility *x* of DCBNT in five binary solvents fitting by three model equations, respectively. In terms of solubility for DCBNT in five binary solvents, they all increased with increasing temperature, which indicates that the dissolution process is endothermic. Compared with these three binary solvents, the solubility of DCBNT in ACN + H_2_O increased slowly with temperature, but in BL + H_2_O the solubility was fastest. The order of DCBNT solubility in binary solvents is BL + H_2_O > DMSO + H_2_O > DEF + H_2_O > DMF + H_2_O > ACN + H_2_O.

**Table 3.** Mole fraction solubility *x* of DCBNT in the binary solvent mixtures (volume ratio = 1 : 1) at different temperatures under 101 kPa^a^.

		Apelblat model		polynomial model		Yaws model	
*T* (K)^b^	1000*x*^c^	1000*x*^cal^	RD	1000*x*^cal^	RD	1000*x*^cal^	RD
DMSO + H_2_O							
300.2	0.082	0.102	−0.246	0.103	−0.256	0.102	−0.244
310.5	0.164	0.160	0.020	0.147	0.104	0.160	0.024
315	0.205	0.196	0.046	0.184	0.102	0.195	0.049
320	0.246	0.243	0.012	0.237	0.037	0.242	0.016
326.5	0.328	0.322	0.018	0.327	0.003	0.323	0.015
330.2	0.39	0.378	0.032	0.388	0.005	0.378	0.031
334.6	0.451	0.456	−0.011	0.470	−0.042	0.456	−0.011
337.6	0.512	0.519	−0.012	0.532	−0.039	0.519	−0.014
340.6	0.574	0.591	−0.025	0.599	−0.044	0.590	−0.028
345	0.697	0.710	−0.017	0.705	−0.011	0.711	−0.020
347.9	0.82	0.801	0.023	0.780	0.049	0.802	0.022
ACN + H_2_O							
298.8	0.041	0.047	−0.140	0.046	−0.122	0.047	−0.136
306.5	0.062	0.063	−0.013	0.062	0	0.063	−0.013
309.5	0.077	0.070	0.087	0.070	0.091	0.070	0.086
313	0.082	0.080	0.023	0.080	0.024	0.080	0.022
322.1	0.113	0.112	0.012	0.114	−0.009	0.112	0.009
331.9	0.154	0.158	−0.025	0.161	−0.045	0.158	−0.028
336.7	0.185	0.187	−0.008	0.188	−0.016	0.187	−0.012
342	0.226	0.224	0.012	0.220	0.027	0.223	0.012
BL + H_2_O							
298	0.125	0.078	0.376	0.122	0.024	0.079	0.367
306	0.167	0.139	0.168	0.153	0.084	0.139	0.170
311	0.208	0.188	0.096	0.194	0.067	0.190	0.088
317	0.25	0.267	−0.068	0.264	−0.056	0.269	−0.072
323.4	0.292	0.373	−0.277	0.365	−0.25	0.374	−0.279
329.7	0.5	0.502	0.043	0.491	0.018	0.500	−0.021
332.9	0.542	0.577	−0.004	0.564	−0.041	0.575	−0.061
337	0.733	0.682	−0.065	0.670	0.086	0.680	0.074
346	1	0.943	0.057	0.937	0.063	0.941	0.059
352.2	1.108	1.145	−0.033	1.154	−0.042	1.150	−0.033
353	1.167	1.171	−0.003	1.182	−0.013	1.170	−0.005
DEF + H_2_O							
299.4	0.034	0.080	−1.353	0.065	−0.912	0.079	−1.324
302.7	0.059	0.094	−0.593	0.083	−0.407	0.094	−0.593
303.9	0.118	0.100	0.110	0.091	0.229	0.100	0.153
308.7	0.153	0.126	0.153	0.122	0.203	0.126	0.176
314.2	0.189	0.162	0.176	0.165	0.127	0.162	0.143
319.5	0.224	0.204	0.143	0.213	0.049	0.204	0.089
323.3	0.248	0.239	0.089	0.251	−0.012	0.240	0.032
327.7	0.283	0.286	0.036	0.299	−0.057	0.287	−0.014
334.6	0.354	0.373	−0.011	0.383	−0.082	0.374	−0.056
338.8	0.413	0.436	−0.054	0.439	−0.063	0.436	−0.056
341.8	0.472	0.485	−0.056	0.482	−0.021	0.485	−0.028
346.2	0.59	0.565	0.042	0.548	0.071	0.564	0.044
DMF + H_2_O							
300	0.033	0.073	−1.212	0.058	−0.758	0.072	−1.183
304	0.067	0.089	−0.328	0.078	−0.164	0.088	−0.318
307.4	0.117	0.105	0.103	0.098	0.162	0.105	0.109
312.9	0.167	0.135	0.192	0.134	0.198	0.135	0.193
323	0.233	0.208	0.107	0.216	0.073	0.208	0.108
330	0.283	0.273	0.035	0.285	−0.007	0.274	0.033
336.8	0.333	0.350	−0.051	0.362	−0.087	0.351	−0.053
339.2	0.383	0.382	0.003	0.366	0.044	0.382	0.004
345.3	0.433	0.468	−0.081	0.391	0.097	0.468	−0.080
351.3	0.549	0.566	−0.031	0.557	−0.015	0.566	−0.030
352	0.616	0.578	0.062	0.567	0.080	0.577	0.063

^a^Standard uncertainties *u* are *u*(*T*) = 0.01 K, *u*(*P*) = 3 kPa.

^b^The estimated relative standard uncertainty of the temperature is *ur*(*T*) = 0.003.

^c^*x* is the experimental solubility data of DCBNT.

**Table 4.** Regressed parameters, RAD and RMSD for the solubility of DCBNT in binary (volume ratio = 1 : 1) solvents with three models at pressure *P* = 101 kPa^a^.

equation	solvent	A^b^	B^b^	C^b^	*R*^2^^c^	RAD^d^	RMSD^e^
Apelblat model	DMSO + H_2_O	−155.814	3179.633	23.846	0.9966	0.042	1.20 × 10^−5^
	ACN + H_2_O	−65.943	−477.500	10.100	0.9949	0.040	3.68 × 10^−6^
	BL + H_2_O	381.593	−23 020.226	−55.080	0.9848	0.111	4.12 × 10^−5^
	DEF + H_2_O	71.139	−7468.460	−9.756	0.9717	0.228	2.47 × 10^−5^
	DMF + H_2_O	107.910	−9153.307	−15.240	0.9755	0.200	2.56 × 10^−5^
polynomial model	DMSO + H_2_O	23.695	−0.159	2.67 × 10^−4^	0.9901	0.063	1.98 × 10^−5^
	ACN + H_2_O	4.475	−0.031	5.517 × 10^−5^	0.9920	0.042	4.65 × 10^−6^
	BL + H_2_O	28.938	−0.195	3.285 × 10^−4^	0.9863	0.068	3.90 × 10^−5^
	DEF + H_2_O	8.561	−0.062	1.117 × 10^−4^	0.9706	0.186	2.51 × 10^−5^
	DMF + H_2_O	7.823	−0.056	1.013 × 10^−4^	0.9751	0.153	2.67 × 10^−5^
Yaws model	DMSO + H_2_O	24.505	−12 122.423	1.225	0.9965	0.043	1.19 × 10^−5^
	ACN + H_2_O	13.944	−6657.137	470 401.797	0.9948	0.040	3.70 × 10^−4^
	BL + H_2_O	−12.560	12 645.584	−2.879	0.9844	0.112	4.21 × 10^−5^
	DEF + H_2_O	5.707	−294.364	−650 820.894	0.9718	0.226	2.46 × 10^−5^
	DMF + H_2_O	2.638	1524.471	−931 513.005	0.9756	0.198	2.56 × 10^−5^

^a^The standard uncertainty is *u*(*P*) = 3 kPa.

^b^A, B and C refer to the parameters of these models.

^c^*R*^2^ is the correlation coefficient.

^d^RAD is the average relative deviation.

^e^RMSD represents the root-mean-square deviation.

From tables 3 and 4, we can also find that the values of correlation coefficient (*R*^2^) are all close to 1, which shows that the values obtained by three models agreed well with the experimental values as well as the RDs. However, the polynomial model is superior to the Apelblat model and the Yaws model in the correlation results of DMSO + H_2_O, DEF + H_2_O, DMF + H_2_O and ACN + H_2_O. At the same time, the correlation results of BL + H_2_O show that the Yaws model is superior to the Apelblat equation and the polynomial model. Besides, the values of RADs and RMSDs of the polynomial model, the Apelblat model and the Yaws model are basically consistent. From RMDS, the values associated with the Apelblat model in DMSO + H_2_O, ACN + H_2_O, BL + H_2_O, DEF + H_2_O and DMF + H_2_O (1.20 × 10^−5^, 3.68 × 10^−6^, 4.12 × 10^−5^, 2.47 × 10^−5^ and 2.56 × 10^−5^) were better than those associated with the polynomial model (1.98 × 10^−5^, 4.65 × 10^−6^, 3.90 × 10^−5^, 2.51 × 10^−5^ and 2.67 × 10^−5^) and the Yaws model (1.19 × 10^−5^, 3.70 × 10^−6^, 4.21 × 10^−5^, 2.46 × 10^−5^ and 2.56 × 10^−5^), indicating that the deviation between the calculated value and the experimental value obtained by the Apelblat model is smaller. In conclusion, the Apelblat model, the Yaws model and the polynomial model can accurately correlate the solubility of DCBNT in binary solvents composed of organic solvents and water. Therefore, we believe that these three models can be used to correlate the solubility data of DCBNT in further study of DCBNT.

In sum, the solubility of DCBNT in all solvents increased with increasing temperature, showing that the solubility of DCBNT in various solvents is closely related to temperature. Likewise, the composition of solvent has a great influence on the solubility of DCBNT. These results provide a theoretical basis for the thermodynamic analysis of the dissolution process.

### 4.2. Thermodynamic properties of DCBNT in solution

The thermodynamic properties for DCBNT in different solvents were described through the standard dissolution enthalpy, standard dissolution entropy and Gibbs free energy, which were calculated according to the modified Apelblat model equation [7,26]. The equation for standard molar dissolution enthalpy (Δ*H*_sol_) is as follows:

4.4 $$\Delta H_{\mathrm{sol}}=-R\times \left(\frac{\partial \text{ln}x}{\partial (1/T)}\right),$$

where Δ*H*_sol_ is the standard molar enthalpy dissolution; *R* is the gas constant; *x* is the mole fraction solubility of DCBNT; and *T* is the solution temperature (K).

From equations (3.1) and (4.4), equation (4.5) can be obtained as follows:

4.5 $$\Delta H_{\mathrm{sol}}=RT\left(C-\frac{B}{T}\right).$$

The equation of mole Gibbs free energy is shown as follows:

4.6 $$\Delta G_{\mathrm{sol}}=-RT\ln x.$$

The molar entropy of dissolution can be obtained through the standard molar dissolution enthalpy and mole Gibbs free energy, as shown in the following equation [27,28]:

4.7 $$\Delta S_{\mathrm{sol}}-\frac{\Delta H_{\mathrm{sol}}-\Delta G_{\mathrm{sol}}}{T}.$$

The final functions were obtained as follows:

4.8 $$\Delta H_{\mathrm{sol}}=RT\left(C-\frac{B}{T}\right),$$

4.9 $$\Delta S_{\mathrm{sol}}=R(A+C+C\text{ln}T)$$

4.10 $$\;\text{and}\;\Delta G_{\mathrm{sol}}=-RT\left(A+\frac{B}{T}+C\text{ln}T\right),$$

where *A*, *B* and *C* are the parameters gained from the modified Apelblat model (tables 2 and 4). The mean temperature *T* was defined by the following equation for minimizing the error propagation [29,30]:

4.11 $$T=\frac{N}{\sum (1/T_{i})},$$

where *N* is the number of temperature points measured in the experiment.

The following equations are used to compare the relative contribution of enthalpy (%*ζ*_H_) and entropy (%*ζ*_TS_) to the dissolution of DCBNT:

4.12 $$\text{\%}\zeta_{H}=\frac{\left|\Delta H_{\mathrm{sol}}\right|}{\left|\Delta H_{\mathrm{sol}}|+|T\Delta S_{\mathrm{sol}}\right|}\times 100$$

and

4.13 $$\text{\%}\zeta_{\mathrm{TS}}=\frac{\left|T\Delta S_{\mathrm{sol}}\right|}{\left|\Delta H_{\mathrm{sol}}|+|T\Delta S_{\mathrm{sol}}\right|}\times 100.$$

The variables Δ*H*_sol_, Δ*S*_sol_, Δ*G*_sol_, %*ζ*_H_ and %*ζ*_TS_ were calculated from equations (4.4) to (4.13) and summarized in tables 5 and 6. Δ*H*_sol_ and Δ*G*_sol_ in pure and binary solvents are all positive, indicating that the dissolution process of DCBNT in all tested solvents is endothermic [31,32].

**Table 5.** Thermodynamic properties for the dissolution of DCBNT in pure solvents.

solvents	*T*	Δ*H*_sol_ (kJ mol^−1^)^a^	Δ*S*_sol_ (J mol^−1^ K^−1^)^b^	Δ*G*_sol_ (kJ mol^−1^)^c^	%*ζ*_H_^d^	%*ζ*_TS_^e^
DMSO	322.6	31.64	58.77	12.68	62.53	37.47
H_2_O	322.0	31.01	64.24	23.89	59.99	40.01
DEF	324.5	33.25	33.64	22.33	75.28	24.72
BL	330.2	26.25	4.51	24.76	94.63	5.37

^a^The solution enthalpy of DCBNT.

^b^The solution entropy of DCBNT.

^c^The Gibbs free energy of DCBNT dissolution in solution.

^d^The relative contributions of enthalpy to dissolution of DCBNT.

^e^The relative contributions of entropy to dissolution of DCBNT.

**Table 6.** Thermodynamic properties for the dissolution of DCBNT in binary solvents.

solvents	*T*	Δ*H*_sol_ (kJ mol^−1^)^a^	Δ*S*_sol_ (J mol^−1^ K^−1^)^b^	Δ*G*_sol_ (kJ mol^−1^)^c^	%*ζ*_H_^d^	%*ζ*_TS_^e^
DMSO + H_2_O	327.4	38.47	50.95	21.79	69.75	30.25
ACN + H_2_O	319.4	30.79	19.94	24.42	82.86	17.14
BL + H_2_O	326.9	41.69	63.34	20.98	66.82	33.18
DEF + H_2_O	321.0	36.06	42.21	22.51	72.69	27.31
DMF + H_2_O	326.4	34.74	37.07	22.64	74.17	25.83

^a^The solution enthalpy of DCBNT.

^b^The solution enthalpy of DCBNT.

^c^The Gibbs free energy for the solution process of DCBNT.

^d^The relative contributions by enthalpy towards the solution process.

^e^The relative contributions by entropy towards the solution process under the experimental conditions.

The results can be extracted from tables 5 and 6 that the enthalpy and the standard Gibbs free energy of DCBNT are positive in both studied pure solvent and binary solvents, indicating that the solution process of DCBNT in all of these solvents is endothermic. The values of Δ*S*_sol_ were positive, indicating that it is an entropy-driven dissolution process. Moreover, by comparing %*ζ*_H_ with %*ζ*_TS_, it can be concluded that the dissolution enthalpy is the main contributor of Gibbs free energy in the dissolution process of DCBNT, because all values of %*ζ*_H_ are ≥62.98%. In addition, Δ*G*_sol_ represents the minimum energy that is required to dissolve DCBNT under the experimental conditions. As shown in tables 5 and 6, the Δ*G*_sol_ value in DMSO + H_2_O and DEF + H_2_O is higher than that in the corresponding pure solvents, which is exactly the opposite in BL. So, the solubility of DCBNT is better in DMSO and DEF than in their binary solvents, but is better in BL + H_2_O than in BL.

## 5. Conclusion

The solubility data of DCBNT in pure and binary solvents were measured at different temperatures from 290 to 360 K by the dynamic method. We can make the following conclusions: (i) the solubilities of DCBNT in all solutions increased with an increasing temperature; (ii) the solubility of DCBNT in DMSO is nearly 100 times higher than that of water and almost insoluble in DMF, methanol, ethanol, acetone, chloroform, dioxane, acetonitrile and trichloromethane, and the solubility of DCBNT in pure solvents is not only related to the polarity of solvent, but also related to other factors; (iii) the solubility data could be successfully correlated using the modified Apelblat model, the Yaws model and the polynomial model, and the fitting result of the three models is basically the same; and (iv) the thermodynamic properties for the solution process including Gibbs energy, dissolution enthalpy and the dissolution entropy were obtained by the Apelblat analysis and the standard Gibbs free energy shows that the dissolving process of DCBNT in all of these solvents is endothermic, and the enthalpy is a main contributor to the dissolution process of DCBNT.

## Supplementary Material

Reviewer comments

## Data accessibility

All the data in this investigation have been reported in the paper and are freely available.
